# Abnormal organization of white matter networks in patients with subjective cognitive decline and mild cognitive impairment

**DOI:** 10.18632/oncotarget.10601

**Published:** 2016-07-13

**Authors:** Xiao-Ni Wang, Yang Zeng, Guan-Qun Chen, Yi-He Zhang, Xuan-Yu Li, Xu-Yang Hao, Yang Yu, Meng Zhang, Can Sheng, Yu-Xia Li, Yu Sun, Hong-Yan Li, Yang Song, Kun-Cheng Li, Tian-Yi Yan, Xiao-Ying Tang, Ying Han

**Affiliations:** ^1^ Department of Neurology, XuanWu Hospital of Capital Medical University, Beijing, China; ^2^ School of Life Science, Beijing Institute of Technology, Beijing, China; ^3^ Department of Radiology, XuanWu Hospital of Capital Medical University, Beijing, China; ^4^ Center of Alzheimer's Disease, Beijing Institute for Brain Disorders, Beijing, China

**Keywords:** subjective cognitive decline, amnestic mild cognitive impairment, network, white matter, diffusion tensor imaging, Pathology Section

## Abstract

Network analysis has been widely used in studying Alzheimer's disease (AD). However, how the white matter network changes in cognitive impaired patients with subjective cognitive decline (SCD) (a symptom emerging during early stage of AD) and amnestic mild cognitive impairment (aMCI) (a pre-dementia stage of AD) is still unclear. Here, structural networks were constructed respectively based on FA and FN for 36 normal controls, 21 SCD patients, and 33 aMCI patients by diffusion tensor imaging and graph theory. Significantly lower efficiency was found in aMCI patients than normal controls (NC). Though not significant, the values in those with SCD were intermediate between aMCI and NC. In addition, our results showed significantly altered betweenness centrality located in right precuneus, calcarine, putamen, and left anterior cingulate in aMCI patients. Furthermore, association was found between network metrics and cognitive impairment. Our study suggests that the structural network properties might be preserved in SCD stage and disrupted in aMCI stage, which may provide novel insights into pathological mechanisms of AD.

## INTRODUCTION

Alzheimer's disease (AD), a progressive neurodegenerative disease characterized by memory or other cognitive domain impairments, is the most common type of dementia. Mild cognitive impairment (MCI) is a transient stage between dementia and normal aging. Amnestic MCI (aMCI), a sub-type of MCI, is regarded as a prodromal state of AD with a dementia conversion rate of 10-15% [1]. Unfortunately, there is no therapeutic drug to stop or reverse disease progression, but early intervention may slow down the progression of the disease [2].

Subjective cognitive decline (SCD) refers to those who have complains about decline in memory or other cognitive functions, but perform normally on cognitive screening [3]. Recent investigations also demonstrated that SCD patients show a greater risk developing into MCI or dementia and could predict AD independently [4–7]. Amyloid deposition and cerebrospinal fluid AD profile have been found in SCD patients, indicating that AD accounts for a major of SCD. Similar structural or functional alterations have been found in SCD as AD or MCI patients [8–16]. Thus, people with SCD are at higher risk of AD and might be important in the study and early diagnosis for AD.

Diffusion tensor imaging (DTI) is able to non-invasively measure white matter integrity and fiber connectivity *in vivo*. Previous studies demonstrated widespread white matter impairment (including frontal, parietal, temporal lobes, the corpus callosum) in both MCI and SCD patients [12, 17–21]. While a growing body of evidence emerging from various techniques suggested that AD is a disconnection syndrome and a failure of dynamic network [22–24]. Network analysis based on the white matter network in AD have suggested that both patients and controls present small-world characteristics with high local inter-connectivity and small path lengths, but implied a weakening small-worldness with either abnormal local efficiency or global efficiency in patients [25–30]. Disrupted topological properties of structural network was also found in preclinical AD as compared to normal controls [31]. While how small-word properties change in SCD and aMCI patients as well as the correlation between the alterations and behavior are not clear.

Here, we used DTI tractography and graph theory to construct weighted structural networks for the NC, SCD, and aMCI groups respectively. Then, both global and nodal parameters were compared among these three groups. We hypothesized that network topographical structure might have been disrupted in SCD stage and become more severe in aMCI stage. We hope to provide new insights into AD pathological mechanism and early diagnosis.

## RESULTS

### Demographics and neuropsychological test results

Thirty-six normal controls, 21 SCD patients, and 33 aMCI patients were finally included in this study. 4aMCI, 2SCD and 4 normal controls were excluded due to the failure of image registration from T1-weighted image to MNI template. There were no differences in age, education, and gender among the three groups (all P>0.05). The Mini-Mental State Examination (MMSE) and Montreal Cognitive Assessment (MoCA) scores were significantly lower in the aMCI group than normal controls (NC) or the SCD group. The group effects in Auditory Verbal Learning Test (AVLT) scores were significant, with the best performance in NC, intermediate performance in SCD patients, and worst performance in aMCI patients. (Table 1)

**Table 1:** results of demographic characteristics and neuropsychological test

Characteristic	NC(n=36)	SCD(n=21)	aMCI(n=33)	Test statistic	*P* value
Age(y)	61.8±7.5	62.9±9.2	64.3±9.7	F=0.72	0.488
Education(y)	11.3±4.4	10.7±4.1	9.3±3.7	F=1.97	0.145
Sex(M/F)	12/24	6/15	16/17	x2=2.80	0.246
MMSE	28.1±1.9	27.9+1.5	25.0±3.0	F=18.36	<0.001 bc
MoCA	26.7±2.7	26.0±2.0	19.8±3.8	F=50.64	<0.001 bc
AVLT-I	8.9±1.5	7.8±1.9	5.7±1.5	F=33.84	<0.001abc
AVLT-D	10.3±2.4	7.9±2.4	3.9±2.4	F=61.25	<0.001abc
AVLT-R	12.5±2.1	10.4±2.2	7.9±4.1	F=20.72	<0.001abc

Values are given as mean ±SD

a MCI: amnestic mild cognitive impairment; SCD: subjective cognitive decline; NC:normal control; MMSE: Mini-Mental State Examination; MoCA: Montreal Cognitive Assessment; AVLT: Auditory Verbal Learning Test;

a normal control group and SCD group showed significant differences (P < 0.05)

B normal control group and aMCI group showed significant differences (P < 0.05)

c SCD group and aMCI group showed significant differences (P < 0.05)

### Global topology of the white matter connectome

**Figure 1:**
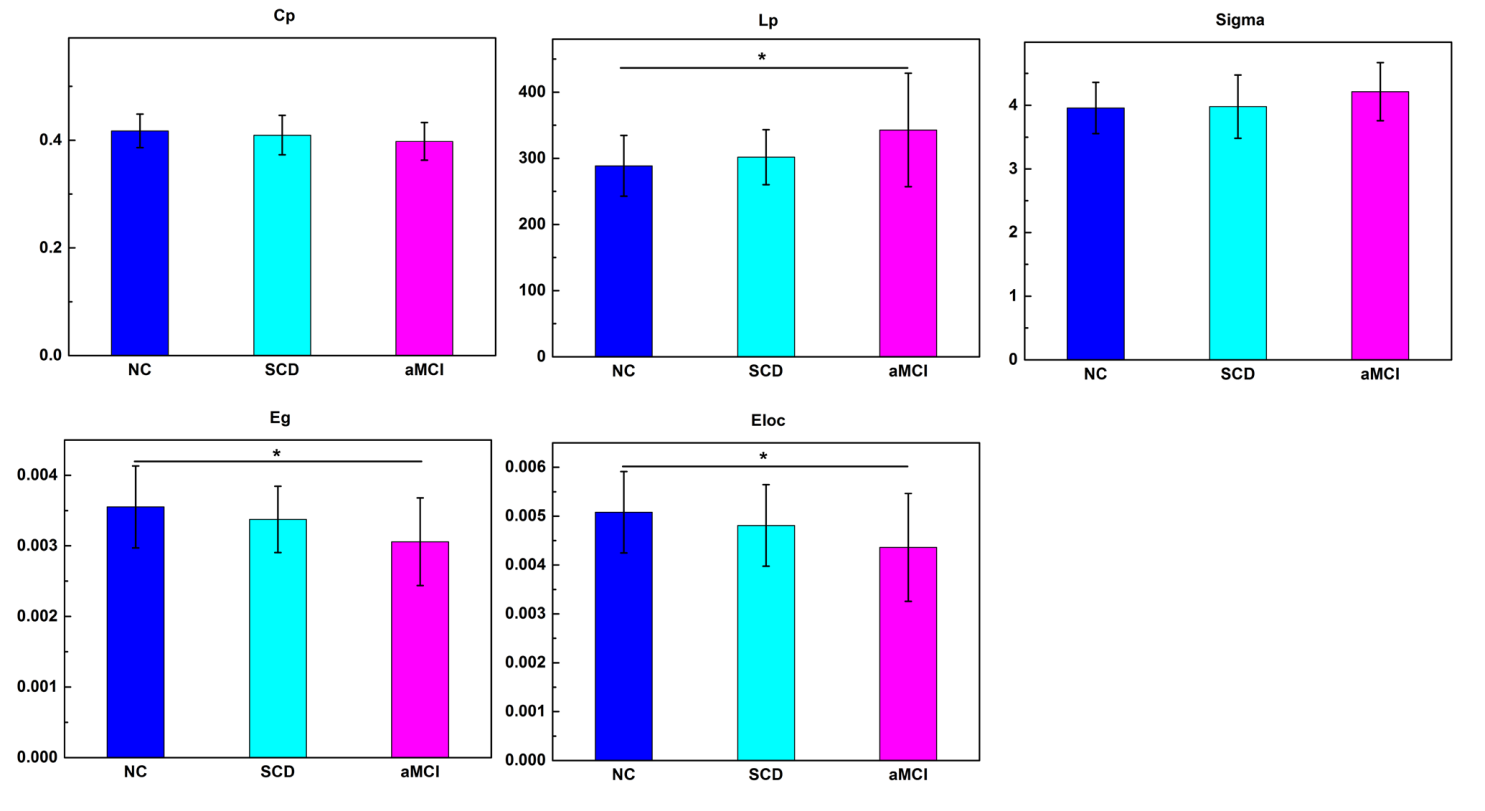
Differences in global measures among the three groups Significant group effects were observed in characteristic path length, global efficiency and local efficiency. Bars and error bars represent mean values and standard deviations, respectively. *P<0.05. **P<0.01. NC, normal controls. Eg, global efficiency. Eloc, local efficiency.

All three groups presented small-world organization (Figure 1). ANCOVAs showed significant group effects on characteristic path length, global efficiency, and local efficiency, with significant decreased global (P=0.002) and local (P=0.007) efficiency, and increased characteristic path length (P=0.002) between aMCI patients and NC, but not between NC and SCD patients or SCD patients and aMCI patients. In addition, there was a linear trend of these altered network properties across three groups (Figure 1).

### Hub regions

**Figure 2:**
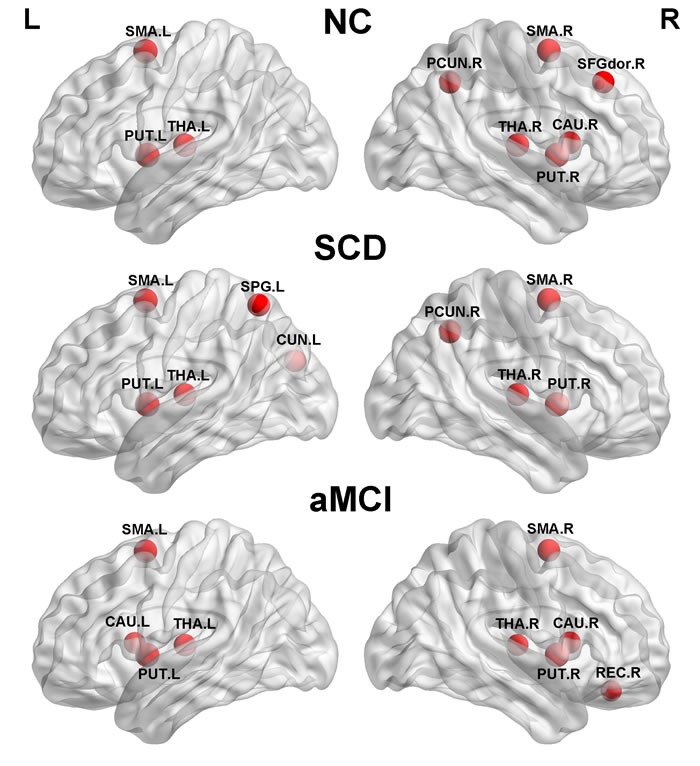
The global network hubs with high betweenness centrality in the normal controls, SCD, and aMCI group The regions were mapped at a lateral view. SMA, supplementary motor area. PUT, putamen. THA, thalamus. PCUN, precuneus. SFGdor, superior frontal gyrus, dorsolateral. CAU, caudate nucleus. SPG, superior parietal gyrus. CUN, cuneus. REC, gyrus rectus. NC, normal controls.

The hub regions are shown in Figure 2. Nine hubs were identified in each groups with six regions in common (bilateral supplementary motor area (SMA), putamen (PUT) and thalamus (THA)). The three other regions in NC were the right pecuneus (PCUN), superior frontal gyrus and dorsolateral (SFGdor) and caudate nucleus (CAU), in SCD were the right PCUN, the left superior parietal gyrus (SPG) and cuneus (CUN), and in aMCI were the bilateral CAU and the right gyrus rectus (REC).

### Differences in betweenness centrality

**Figure 3:**
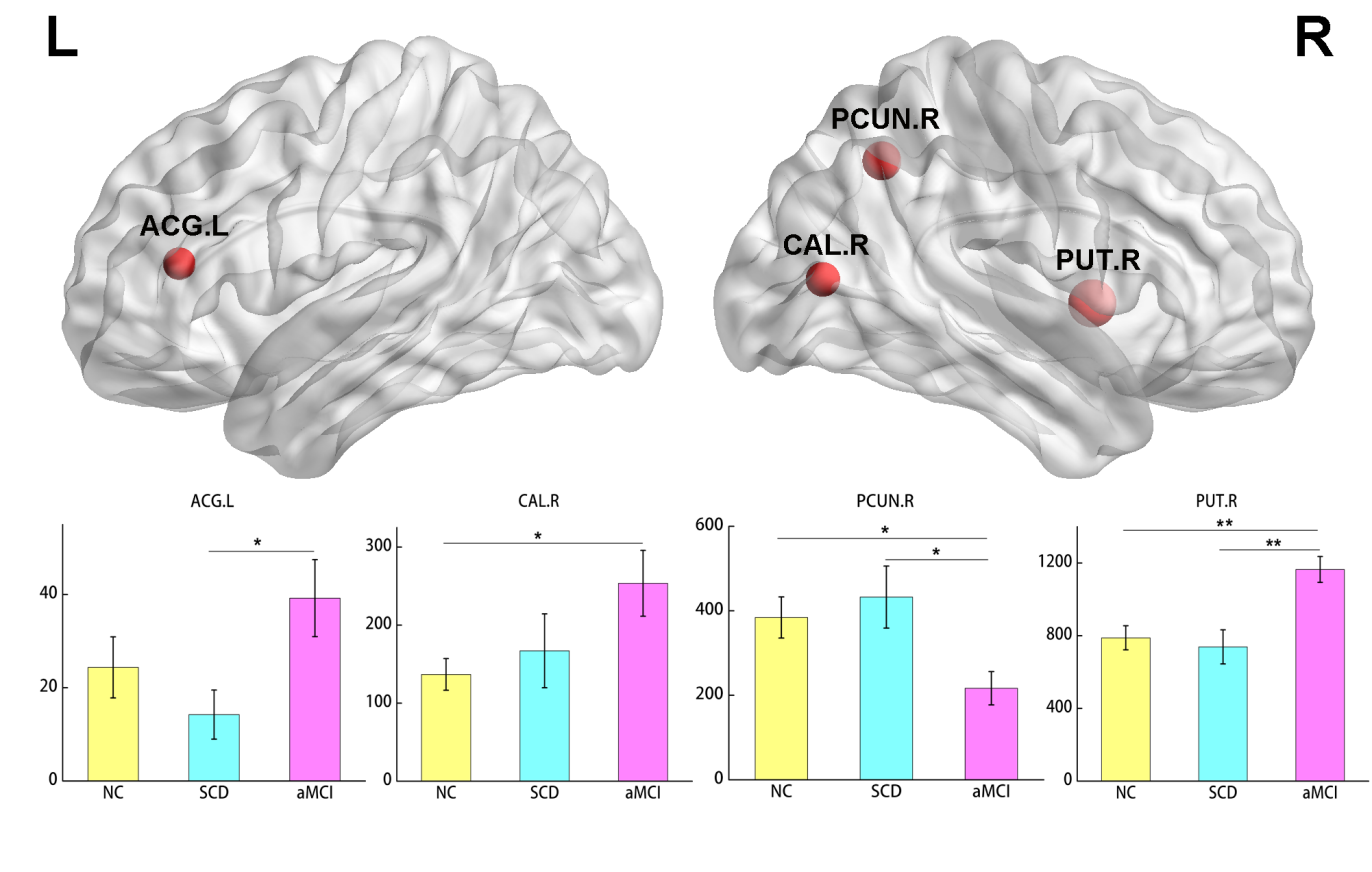
Regions with significant differences in betweenness centrality among the three groups The node size represents the significance of between-group differences. For each node, the bar and error bar indicate the mean value and standard error, respectively. Post hoc tests showed reduced centrality in PCUN and increased centrality in CAL and PUT in aMCI group versus the NC group. Lower centrality was found in PCUN and higher centrality was found in ACG and PUT in aMCI as compared to the SCD patients. *P<0.05. **P<0.01. PCUN, precuneus. CAL, calcarine fissure and surrounding cortex. PUT, putamen. ACG: anterior cingulate and paracingulate gyri. NC, normal controls.

We further localized the nodes with changed betweenness centrality among the three groups (Figure 3). Regions with significant differences across the three groups were located in right PCUN, PUT, calcarine fissure and surrounding cortex (CAL) and left Anterior cingulate (ACG). Post hoc test showed decrease in PCUN and increase in CAL and PUT in aMCI group than NC group. In addition, decrease in PCUN and increase in ACG and PUT were also found in aMCI as compared to the SCD patients.

### Relationship between network metrics and cognition

We further examined the correlation between network metrics and behavior performance (Figure 3). In SCD patients, no correlation between network properties and neuropsychological tests was found. In the aMCI group, the left ACG was positively correlated with scores of MoCA (r=0.37, P=0.044) and AVLT delayed recall (r=0.453, P=0.012). While negative correlation was found between AVLT delayed recall and right PUT in aMCI groups.

**Figure 4:**
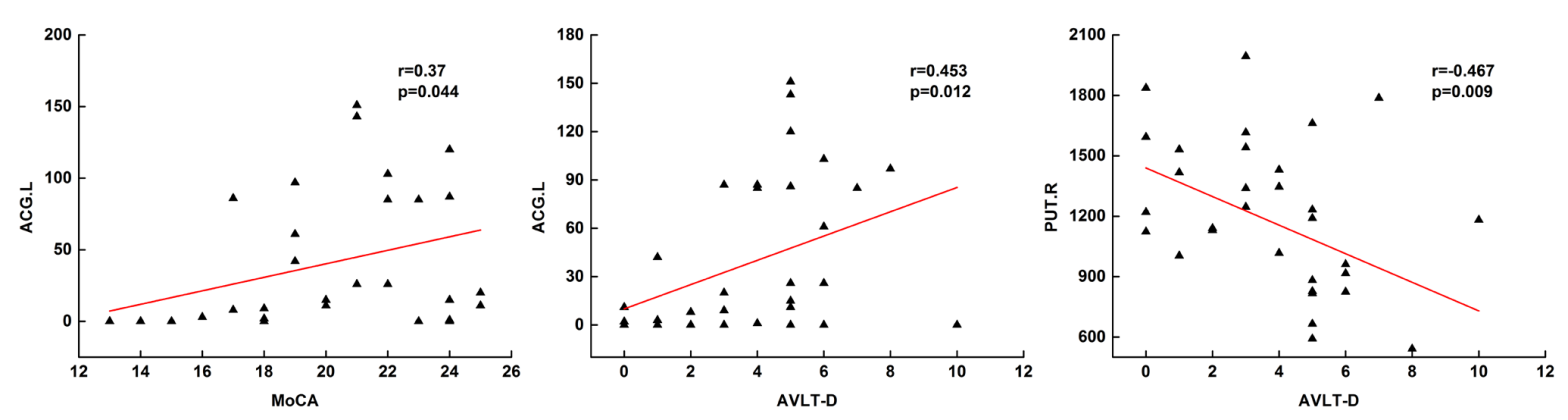
Scatter plots of betweenness centrality and memory performances in aMCI patients

## DISCUSSION

We investigated the architecture of white matter networks in patients with SCD and aMCI. The main findings in our study were as follow: 1) small-world topology in both the normal controls and patients, which is in agreement with previous studies [25–30]; 2) increased path length and decreased efficiency in patients; 3) alterations in regional centrality in patients; 4) correlation between network metrics and behavior.

We demonstrated a lower efficiency in aMCI patients compared to NC, which indicates a less optimal organization on patients. A lower global efficiency as well as a higher Lp found in aMCI patients is in line with previous studies [25–29], which indicates the loss of ability to transmit specialized information rapidly among distant brain regions [32]. While the inconsistency observed between Cp and local efficiency as found in previous studies might be due to the influence of connection strength on Cp [33–35]. The loss of nodes and a more-diffuse impairment pattern would lead to a higher Cp [25, 36]. So both the increment in Cp and reduction in local efficiency indicated inefficient/ineffective information transmission in aMCI patients. A novel finding of this study was that SCD patients had values intermediate to aMCI and NC, suggesting a similar damage pattern of structure network in SCD but milder than aMCI patients. Such tendency has been found repeatedly in other studies by Tract-Based Spatial Statistics [13].

Recent researches have shown that hubs like the precuneus, the medial frontal cortex, the middle occipital and the cingulated gyrus are more vulnerable [37, 38]. We found two hubs (right PCUN and putamen) in our study presented significant alterations in centrality with disease prognosis, especially the right PCUN, which was no more a hub in aMCI patients. The PCUN located in medial posterior parietal cortex is a functional core of default mode network (DMN) and involved in various cognitive process like visual-spatial, self-processing, consciousness and episodic memory [39–40]. Decreased nodal strength was also found in PCUN in previous studies [41]. Evidence also have shown lower FA in PCUN in SCD patients and persons converting to MCI [12, 42]. It may provide implication for memory impairment in patients.

The sub-cortical putamen has been found lower nodal strength in aMCI patients in previous studies [41], but showed an increase in centrality in our study conversely. Such alterations were also found in two non-hubs (ACG and calcarine). Betweenness centrality, measuring the importance of the node for information transmission, is not in parallel with node degree [43]. Previous studies have found non-monotonic changes in AD with higher betweenness centrality in aMCI and mild AD dementia than prior stages. Increased centrality was also found in lingual gyrus, cingulate and lateral occipitotemporal gyrus by sMRI and fMRI [44–45]. We speculated the increase of these nodes might be compensatory for the reduced centrality in PCUN. Further, we found that the centrality in ACG and PUT was correlated with behavior, which indicates the potential of centrality for early diagnosis. While the correlation in ACG was positive, but in PUT was negative. One possible suppose for the discrepancy is that the compensatory mechanism is reserved in PUT with disease progressing but failed for ACG. Despite the aforementioned results, we failed to find any significant difference found between SCD and NC groups, which might because SCD is an earlier stage and the dysfunction is compensatory or because of the limited sample.

We noted that there are still some limitations in our study. First, we employed deterministic tractography to reconstruct structural connectivity as several previous researches [25–27]. While it cannot map out all the fibers accurately, a limitation of tracking crossing fibers and long-distance fibers exists. Second, this study is lack of pathological evidence with PIB-PET or CSF biomarker. Though all subjects went through a series of neuropsychological tests and MRI to exclude other diseases like depression, brain trauma, and vascular dementia, we could not completely ensure that no patients had mixed pathology. Third, this study is cross-sectional and a longitudinal study would be valuable to further explore the network properties of AD. Finally, the sample size now is limited and the results need to be replicated in large samples. In conclusion, our results verified that AD is a disconnection syndrome again. Our findings indicated that white matter network was gradually disrupted as cognitive decline and it has the potential for early diagnosis.

## MATERIALS AND METHODS

### Subjects

A total of 100 right-handed subjects (40 normal controls, 23 SCD, 37 aMCI) were recruited at the memory clinic of Beijing XuanWu Hospital of Capital Medical University in China from January 2011 to March 2015. This study was approved by the Medical Research Ethics Committee and Institutional Review Board of XuanWu Hospital (ClinicalTrials.gov Identifier: NCT02353845) and informed consent was obtained from all subjects.

The patients with aMCI were diagnosed according to Petersen's criteria [1] and National Institute on Aging-Alzheimer's Association criteria for MCI due to AD [46] as following: (a) memory complaint; (b) objective memory impairment - MMSE, MoCA [47], AVLT; (c) near-normal performance on general cognition and preserved daily life activities; (d) the Clinical Dementia Rating (CDR) score of 0.5; (e) failure to meet the criteria for dementia according to the Diagnostic and Statistical Manual of Mental Disorders, fourth edition, revised (DSM-IV) [48]; (f) hippocampal atrophy observed and (h) the Han nationality, right-handed (the Edinburgh handedness scale score > 40 points). The patients with SCD were included as following based on the research criteria for subjective cognitive decline [3]: a) self-experienced decline in memory compared to previous statue (within the last 5 years), which could be confirmed by informants; and (b) normal performances on objective cognitive tests and CDR score = 0. The normal controls were cognitively normal and had a Clinical Dementia Rating (CDR) of 0. Subjects were excluded if they met the following clinical characteristics: (a) those who have a clear history of stroke (Hachinski Ischemic Scale score (HIS)> 7 points); (b) severe depression (Hamilton Depression Rating Scale score (HAMD) > 24 points); (c) cognitive impairment caused by traumatic brain injury; (d) other nervous system diseases, which could cause cognitive impairment; (e) systemic diseases, which could cause cognitive impairment; (f) a history of psychosis or congenital mental growth retardation; and (g) those who cannot corporate with neuropsychological tests or have any contraindication to MRI (Magnetic Resonance Imaging). All of subjects underwent MMSE, MoCA, AVLT, Activity of Daily Living (ADL), HIS, HAMD, and CDR. And all participants underwent a brain MRI.

### MRI Acquisition

The MR images of all patients and normal controls were acquired by a Siemens 3T TrioTim MRI system. T1-weighted MR images were obtained by a 3D magnetization-prepared rapid gradient echo (MPRAGE) with following parameters: Slices = 176, TR = 1900 ms, TE = 2.2 ms, inversion time (TI) = 900 ms, FA =9°, field of view (FOV) = 256×256 mm, acquisition matrix = 256×256, and thickness = 1 mm. DTI data was collected using an echo planar imaging (EPI) sequence with following parameters for three times: in 32 independent, non-collinear directions of a b-value = 1000 s/mm2 and one additional image with no diffusion weighting(b = 0), slices = 60, TR= 11000 ms, TE =98 ms, FA =90°, FOV = 256 mm×256 mm, acquisition matrix= 128×128, and thickness= 2 mm.

### Image Pre-processing and Network Construction

Image pre-processing steps contains: Format conversion of original data (DICOM); The extraction of brain tissue and structure; Realignment; Eddy current and motion artifact correction of diffusion-tensor imaging data; Fractional anisotropy calculation; Diffusion tensor tractography. Tractography was conducted to produce 3-D streamlines representing fiber tract connectivity [49].

WM connectivity was modeled as an weighted network containing 90 nodes, defined by automated anatomic labeling [50]. Each AAL brain region was deemed a node of the brain network. With the usage of PANDA software, we completed the WM deterministic fiber tracking, pairs of nodes were linked if they were interconnected through a certain number of streamlines. In our study, we chose 3 for the threshold value for the streamline numberWe validated the results setting the threshold ranged from 1 to 5 and found the threshold did not significantly effect the results (see Supplementary Material).

We computed the weight of each effective connection between two nodes (i and j) as the product of the connecting fiber number (FN) and mean fractional anisotropy (FA) of the connecting fiber, normalized by the average volume of the two connecting regions to offset the deviation where larger cortical regions are more likely to contain more ‘false’ fibers (${\text{w}}_{\text{ij}}={\text{FN}}^{\text{*}}\text{FA}/\text{volume}$). This weighting method have been employed in several previous diffusion brain network studies [53–54]. Finally, we obtained a symmetric 90 × 90 matrix for each participant from their DTI data. This part of work were done by PANDA toolbox [55], which is on the base of FSL [56] (http://fsl.fmrib.ox.ac.uk/fsl/fslwiki).

### Network parameter calculation

Several network topological properties were used to characterize WM structure network derived from each participant, including: clustering coefficient (Cp), characteristic path length (Lp), small-worldness(Sigma), local efficiency (Eloc), global efficiency (Eg) and nodal betweenness (B_nod_) [32]. In this study, we calculated all these parameter metrics using GRETNA v1.2 (http://www.nitrc.org/projects/gretna), which is a graph-theorectical network analysis toolkit.

For a given network G with N nodes, the clustering coefficient(Cp) and the characteristic path length(Lp) were defined by Watts and Strogatz [57] as:

$$\text{Cp(G)}=\frac{\text{1}}{\text{N}}\sum_{\text{i}\in \text{G}}\frac{{\text{E}}_{\text{i}}}{{\text{D}}_{\text{nod}}{\text{(i)(D}}_{\text{nod}}\text{(i)}-\text{1})/\text{2}}$$

Where D_nod_(i) is the degree of a node i. E_i_ is the number of edges in G_i_, which is the subgraph composed of the adjacent nodes of a node i.

$$\text{Lp(G)}=\frac{1}{\frac{1}{\text{N(N}-\text{1)}}(\sum_{\text{j}\neq \text{i}\in \text{G}}\frac{1}{{\text{L}}_{\text{IJ}}})}$$

Where L_ij_ is the shortest path length between nodes i and j. To figure out the small-worldness parameter, the values of Cp and Lp were normalized by compared with those of 100 random networks $\left(\gamma \;=\;{\text{Cp}}^{\text{real}}/{\text{Cp}}^{\text{rand}}\;\text{and}\;\lambda \;=\;{\text{Lp}}^{\text{real}}/{\text{Lp}}^{\text{rand}}\text{,}\text{Sigma}=\gamma /\lambda \right)$.

A network is said to have small-worldness, if it has similar Lp but higher Cp than random networks. In other words, a small-word network has a normalized clustering coefficient (γ ≈ 1) and a normalized path length (λ > 1).

The global efficiency and local efficiency of G were defined by Latora and Marchiori [58] as:

$$\text{Eg}\left(\text{G}\right)\;=\;\frac{1}{\text{N}(\text{N}-1)}\sum_{\text{j}\neq \text{i}\in \text{G}}\frac{1}{L_{\text{ij}}}$$

and

$$\text{Eloc}\left(\text{G}\right)\;=\;\frac{1}{\text{N}}\sum_{\text{i}\in \text{G}}\text{Eg}\left({\text{G}}_{\text{I}}\right)$$

Where $\text{Eg}\left({\text{G}}_{\text{I}}\right)$is the global efficiency of- G_i-_, which is the subgraph consisting of the adjacent nodes of a node i.

The nodal matrices measure the importance of all nodes in the network. Betweenness centrality evaluates the contribution of a node on the communication for other nodes. The betweenness centrality of a node i was defined by Freeman [59] as:

$${\text{B}}_{\text{nod}}\left(\text{i}\right)\;=\;\sum_{\text{j}\neq \text{i}\neq \text{k}\in \text{G}}\frac{\delta_{\text{jk}}\left(\text{i}\right)}{\delta_{\text{jk}}}$$

Where $\delta_{\text{jk}}$is the number of shortest paths from a node j to a node k, and $\delta_{\text{jk}}\left(\text{i}\right)$ is the number of shortest paths from a node j to a node k via a node i within the network G.

The top 10% hubs of nodal betweenness were depicted by Brainnet Viewer toolbox [60].

### Statistical Analysis

Statistical analysis was performed with software SPSS v20.0. Group differences in age, years of education and neuropsychological scores were examined with one-way ANOVA. Post hoc pairwise t test with Bonferroni correction for multiple comparison was performed if ANOVA yielded significant results (P <0.05). Sex data were examined with a Pearson chi-square test. For group effects in global and regional network measures, comparisons were performed among 3 groups using one-way ANOVA with post hoc pairwise t tests with Bonferroni correction, when P <0.05.

Finally, we investigated the relationship between network metrics and behavior by partial correlation analysis with age, gender and education as covariates. To identify the correlation between neuropsychological test scores with specific brain regions, the nodes with significant group differences were performed.

## SUPPLEMENTARY MATERIALS TABLE


