# Supplementary material for: Abnormal organization of white matter networks in patients with subjective cognitive decline and mild cognitive impairment
Source: Oncotarget. 2016 Jul 13;7(31):48953–62. doi: 10.18632/oncotarget.10601 (PMC5226483; doi:10.18632/oncotarget.10601)
Supplement: Supplementary file 1 [file oncotarget-07-48953-s001.pdf]

Abnormal organization of white matter networks in patients with subjective cognitive decline and mild cognitive impairment

Supplementary Material

Supp Table 1: Comparison of global measures with FN ranged from 1 to 5

FN threshold	Groups	Cp	Lp	Sigma	Eg	Eloc
1	aMCI	0.45±0.03	340.33±81.93	3.69±0.45	0.0031±0.00062	0.0044±0.00103
	SCD	0.46±0.03	300.30±40.60	3.42±0.42	0.0034±0.00047	0.0048±0.00077
	NC	0.46±0.03	289.10±44.65	3.47±0.35	0.0035±0.00057	0.0050±0.00080
2	aMCI	0.42±0.03	341.38±83.38	3.93±0.46	0.0031±0.00063	0.0044±0.00106
	SCD	0.43±0.03	300.76±40.86	3.70±0.49	0.0034±0.00047	0.0048±0.00075
	NC	0.44±0.03	289.47±44.84	3.74±0.38	0.0035±0.00057	0.0051±0.00080
3	aMCI	0.40±0.04	342.78±85.62	4.21±0.46	0.0031±0.00062	0.0044±0.00110
	SCD	0.41±0.04	301.66±41.67	3.98±0.50	0.0034±0.00047	0.0048±0.00083
	NC	0.42±0.03	288.69±45.95	3.96±0.40	0.0036±0.00058	0.0051±0.00083
4	aMCI	0.38±0.04	345.82±87.81	4.40±0.55	0.0030±0.00063	0.0043±0.00116
	SCD	0.39±0.04	302.80±42.90	4.16±0.56	0.0034±0.00048	0.0048±0.00083
	NC	0.40±0.03	291.19±45.61	4.20±0.44	0.0035±0.00057	0.0050±0.00085
5	aMCI	0.36±0.05	348.76±90.94	4.58±0.62	0.0030±0.00064	0.0042±0.00120
	SCD	0.36±0.04	304.05±43.25	4.32±0.61	0.0034±0.00048	0.0047±0.00092
	NC	0.38±0.03	292.68±45.88	4.36±0.44	0.0035±0.00057	0.0049±0.00089

Adjacent groups show significant differences in network properties are highlighted in shaded cells.

FN: fiber number; NC: normal controls; Eg: global efficiency; Eloc: local efficiency;
